# Supplementary material for: Superinfection and the evolution of an initial asymptomatic stage
Source: R Soc Open Sci. 2021 Jan 27;8(1):202212. doi: 10.1098/rsos.202212 (PMC7890506; doi:10.1098/rsos.202212)
Supplement: Electronic Supplementary Material for: Superinfection and the evolution of an initial asymptomatic stage [file rsos202212supp1.pdf]

Electronic Supplementary Material for: *Superinfection and the evolution of an initial asymptomatic stage*

Chadi M. Saad-Roy[*],[†] Bryan T. Grenfell[‡],[§],[¶] Simon A. Levin[‡] Lorenzo Pellis[||],[**]
Helena B. Stage[||] P. van den Driessche[††] Ned S. Wingreen[*] ,[‡‡]

December 3, 2020

Expanded Results

Since $\widehat{I}_2 = \frac{\nu_1}{\nu_2+\delta}\widehat{I}_1$, and letting $\gamma[\lambda] = \alpha_1[\lambda] + \frac{\nu_1[\lambda]}{\nu_2+\delta}\alpha_2$, it follows from (3) that

$$\mathcal{R}_0[\lambda,\lambda^*] = \left(\frac{\alpha_1[\lambda^*]}{\nu_1[\lambda^*]+\delta+\sigma\gamma[\lambda]\widehat{I}_1[\lambda]} + \frac{\nu_1[\lambda^*]}{\nu_1[\lambda^*]+\delta+\sigma\gamma[\lambda]\widehat{I}_1[\lambda]}\frac{\alpha_2}{\nu_2+\delta}\right)\left(\widehat{S}[\lambda]+\sigma\widehat{I}_1[\lambda]\right), \tag{6}$$

and it can also be checked that $\mathcal{R}_0[\lambda,\lambda] = 1$, which is biologically reasonable.

As defined in the main text, $k = \frac{\alpha_2}{\nu_2+\delta} > 0$ and $\tau = \frac{1}{\nu_2+\delta} + \frac{\nu_2}{\nu_2+\delta}\frac{1}{\mu+\delta}$. We let $\mathcal{B}_1 = k + \frac{\sigma k(k-1)}{\tau\delta}$ and $\mathcal{B}_2 = k + \frac{[(\sigma k(k-1)(\nu_{1\infty}+\delta))/\delta]}{\tau\nu_{1,\infty}+1-k\sigma}$.

Consider the inequality

$$\left((\alpha_{1,0}-k\delta) - \frac{b_1b_2}{c_1c_2}(\nu_{1,0}+\delta)\right) > \sigma\left(\frac{b_1b_2}{c_1c_2}+k\right)\left(\frac{\alpha_{1,0}+(k-1)\nu_{1,0}-\delta}{1+\tau\nu_{1,0}}\right), \tag{7}$$

which is a key determinant of evolutionary dynamics in our model (see Remark 1, Theorems 2, 3, and 4). The evolutionary dynamics depend upon the value of the superinfection parameter (σ), and the evolutionary dynamics landscape, as a function of the transmission decay exponent (b_2) and the fully latent transmission rate times the average lifespan ($\alpha_{1,\infty}/\delta$), transitions as σ varies. For the remainder of this section, we assume that $b_2 > c_2$. First, suppose that $\sigma < \frac{1-\tau\delta}{k}$, which implies that $\mathcal{B}_2 < \mathcal{B}_1$. Then, if $\frac{\alpha_{1,\infty}}{\delta} < \mathcal{B}_2$, there is a unique ESS at zero latency. If $\mathcal{B}_2 < \frac{\alpha_{1,\infty}}{\delta}$ and (7) does not hold, there is at least one repellor that is a fitness minimum, and leads to alternative stable states at zero and infinite latency (Theorems 2-3, *Electronic Supplementary*

[*]Lewis-Sigler Institute for Integrative Genomics, Princeton University, NJ, USA
[†]Correspondence to: csaadroy@princeton.edu
[‡]Department of Ecology and Evolutionary Biology, Princeton University, Princeton, NJ, USA
[§]Princeton School of Public and International Affairs, Princeton University, Princeton, NJ, USA
[¶]Division of International Epidemiology and Population Studies, Fogarty International Center, National Institutes of Health, Bethesda, MD, USA
[||]Department of Mathematics, University of Manchester, Manchester, UK
[**]The Alan Turing Institute, London
[††]Department of Mathematics and Statistics, University of Victoria, Victoria, BC, Canada
[‡‡]Department of Molecular Biology, Princeton University, Princeton, NJ, USA

Material). This repellor is unique if $\frac{\alpha_{1,\infty}}{\delta} > \mathcal{B}_1$ (Theorem 3, *Electronic Supplementary Material*). Otherwise, if (7) holds, then there is a local ESS at infinite latency, which is unique (Theorems 2-3, *Electronic Supplementary Material*).

Suppose the superinfection parameter is such that $\frac{1+\nu_{1,\infty}\tau}{k} > \sigma > \frac{1-\tau\delta}{k}$, which gives that $\mathcal{B}_1 < \mathcal{B}_2$. If $\frac{\alpha_{1,\infty}}{\delta} < \mathcal{B}_1$, there is again a unique ESS at zero latency (Theorems 2-3, *Electronic Supplementary Material*). If $\mathcal{B}_1 < \frac{\alpha_{1,\infty}}{\delta} < \mathcal{B}_2$, then there is at least one evolutionarily singular strategy that is convergence stable if (7) does not hold (Theorem 3, *Electronic Supplementary Material*). Since this strategy is a fitness minimum and is hence not evolutionarily stable, this attractor is a branching point and leads to mutual invasibility (Theorem 2, *Electronic Supplementary Material*). Otherwise, if (7) holds, there is a local ESS at zero latency (Theorem 3, *Electronic Supplementary Material*). If $\frac{\alpha_{1,\infty}}{\delta} > \mathcal{B}_2$ and (7) does not hold, then there is at least one repellor that is not evolutionarily stable, and there are alternative stable states at zero and infinite latency (Theorems 2-3, *Electronic Supplementary Material*). If (7) holds, then there is a unique ESS at infinite latency (Theorems 2-3, *Electronic Supplementary Material*).

Lastly, suppose that the superinfection is greater than $\frac{1+\tau\nu_{1,\infty}}{k}$, which means that $\mathcal{B}_2 < 0 < \mathcal{B}_1$ (see Remarks 3, 4). Then, there are two possible outcomes. If $\frac{\alpha_{1,\infty}}{\delta} < \mathcal{B}_1$, then there is a unique ESS at zero latency (Theorem 3, *Electronic Supplementary Material*). Otherwise, if $\frac{\alpha_{1,\infty}}{\delta} > \mathcal{B}_1$ and (7) holds, then there is a unique branching point (Theorems 2-3, *Electronic Supplementary Material*). If $\frac{\alpha_{1,\infty}}{\delta} > \mathcal{B}_1$ and (7) does not hold, then there is a local ESS at zero latency along with possible other interior singular strategies (Theorem 3, *Electronic Supplementary Material*).

The addition of infection stages

Suppose that there is a third stage, with force of infection α_3 and recovery rate ν_3, and that hosts in this stage can also superinfect hosts in the first stage. Here, for a unique stable unique endemic equilibrium if $\mathcal{R}_0 > 1$, we assume either zero or complete immunity ($\mu = \infty$, and $\mu = 0$, respectively) [1, 2]. Then, a similar process to derive the basic reproduction number for a pathogen with mutant phenotype gives that

$$\mathcal{R}_0^{[3]}[\lambda,\lambda^*] = \Bigg(\frac{\alpha_1[\lambda^*]}{\nu_1[\lambda^*]+\delta+\sigma(\alpha_1[\lambda]\widehat{I}_1[\lambda]+\alpha_2\widehat{I}_2[\lambda]+\alpha_3\widehat{I}_3[\lambda])} + \frac{\nu_1[\lambda^*]}{\nu_1[\lambda^*]+\delta+\sigma(\alpha_1[\lambda]\widehat{I}_1[\lambda]+\alpha_2\widehat{I}_2[\lambda]+\alpha_3\widehat{I}_3[\lambda])}\frac{\alpha_2}{\nu_2+\delta} + \frac{\nu_1[\lambda^*]}{\nu_1[\lambda^*]+\delta+\sigma(\alpha_1[\lambda]\widehat{I}_1[\lambda]+\alpha_2\widehat{I}_2[\lambda]+\alpha_3\widehat{I}_3[\lambda])}\frac{\nu_2}{\nu_2+\delta}\frac{\alpha_3}{\nu_3+\delta}\Bigg)\left(\widehat{S}[\lambda]+\sigma\widehat{I}_1[\lambda]\right).$$

Letting $\widetilde{k} = k + \frac{\nu_2}{\nu_2+\delta}\frac{\alpha_3}{\nu_3+\delta}$, it follows that $\widetilde{\gamma}[\lambda] = \alpha_1[\lambda] + \nu_1[\lambda]\widetilde{k}$ and so

$$\mathcal{R}_0^{[3]}[\lambda,\lambda^*] = \left(\frac{\alpha_1[\lambda^*]}{\nu_1[\lambda^*]+\delta+\sigma\widetilde{\gamma}[\lambda]\widehat{I}_1[\lambda]} + \frac{\nu_1[\lambda^*]\widetilde{k}}{\nu_1[\lambda^*]+\delta+\sigma\widetilde{\gamma}[\lambda]\widehat{I}_1[\lambda]}\right)\left(\widehat{S}[\lambda]+\sigma\widehat{I}_1[\lambda]\right).$$

Since $\widetilde{k}$ replaces k and this parameter is independent of latency, it follows that the qualitative evolutionary dynamics of latency are the same in this model as in the model with only two stages, with $\widetilde{k}$ replacing k in threshold values. Furthermore, the average time a host cannot get superinfected (τ) is replaced by $\widetilde{\tau} = \frac{1}{\nu_2+\delta} + \frac{\nu_2}{\nu_2+\delta}\frac{1}{\nu_3+\delta} + \frac{\nu_2}{\nu_2+\delta}\frac{\nu_3}{\nu_3+\delta}\frac{1}{\delta+\mu}$.

A similar observation holds if there exists an infection stage I_0 previous to I_1 and I_2 with force of infection α_0 and ν_0 that cannot get superinfected. Then, the basic reproduction of an new mutant can be written as

$$\mathcal{R}_0^{[0]}[\lambda,\lambda^*] = \left(\frac{\alpha_0}{\nu_0+\delta} + \frac{\nu_0}{\nu_0+\delta}\left(\frac{\alpha_1[\lambda^*]}{\nu_1[\lambda^*]+\delta+\sigma\gamma[\lambda]\widehat{I}_1[\lambda]} + \frac{\nu_1[\lambda^*]k}{\nu_1[\lambda^*]+\delta+\sigma\gamma[\lambda]\widehat{I}_1[\lambda]}\right)\right)\left(\widehat{S}[\lambda]+\sigma\widehat{I}_1[\lambda]\right),$$

which is a linear transformation on the dependence of λ^*, *i.e.* $\mathcal{R}_0^{[0]}[\lambda,\lambda^*] = f[\lambda] + c\mathcal{R}_0[\lambda,\lambda^*]$, and thus the qualitative dynamics do not change.

Evolutionary dynamics in the presence of coexistence

If a branching point exists, then by definition there is a region of mutual invasibility. If there is a region where two types can coexist, then this section presents a framework to analyse the evolutionary dynamics after branching. At such a coexistence region, if we denote type 1 with strategy λ_1 and type 2 with strategy λ_2, then the system follows the following set of coupled ordinary differential equations:

$$\frac{dS}{dt} = \delta - (\alpha_{1,2}I_{1,2} + \alpha_2 I_{2,2} + \alpha_{1,1}I_{1,1} + \alpha_2 I_{2,1})S - \delta S + \mu R_1 + \mu R_2,$$

$$\frac{dI_{1,1}}{dt} = (\alpha_{1,1}I_{1,1} + \alpha_2 I_{2,1})(S + \sigma I_{1,2}) - (\nu_1+\delta)I_{1,1} - \sigma(\alpha_{1,2}I_{1,2} + \alpha_2 I_{2,2})I_{1,1},$$

$$\frac{dI_{2,1}}{dt} = \nu_{1,1}I_{1,1} - (\nu_2+\delta)I_{2,1},$$

$$\frac{dR_1}{dt} = \nu_2 I_{2,1} - (\delta+\mu)R_1,$$

$$\frac{dI_{1,2}}{dt} = (\alpha_{1,2}I_{1,2} + \alpha_2 I_{2,2})(S + \sigma I_{1,1}) - (\nu_{1,2}+\delta)I_{1,2} - \sigma(\alpha_{1,1}I_{1,1} + \alpha_2 I_{2,1})I_{1,2},$$

$$\frac{dI_{2,2}}{dt} = \nu_{1,2}I_{1,2} - (\nu_2+\delta)I_{2,2},$$

$$\frac{dR_2}{dt} = \nu_2 I_{2,2} - (\delta+\mu)R_2.$$

If there exists an endemic equilibrium $\widehat{E} = (\widehat{S}, \widehat{I}_{1,1}, \widehat{I}_{2,1}, \widehat{R}_1, \widehat{I}_{1,2}, \widehat{I}_{2,2}, \widehat{R}_2)$ that is stable, then the question of further mutation and evolution can be examined in the adaptive dynamics framework. If a mutant λ_m arises, then letting $\alpha_{1,i} = \alpha_1[\lambda_i]$ and $\nu_{1,i} = \nu_1[\lambda_i]$ for $i = 1, 2$, the fractions infected with this mutant are

$$\frac{dI_{1,m}}{dt} = (\alpha_{1,m}I_{1,m} + \alpha_2 I_{2,m})(S + \sigma(I_{1,1}+I_{1,2})) - (\nu_1+\delta)I_{1,m} - \sigma(\alpha_{1,1}I_{1,1} + \alpha_2 I_{2,1} + \alpha_{1,2}I_{1,2} + \alpha_2 I_{2,2})I_{1,m},$$

$$\frac{dI_{2,m}}{dt} = \nu_{1,m}I_{1,m} - (\nu_2+\delta)I_{2,m}.$$

Using the next-generation matrix [3, 4], then

$$\mathcal{R}_0[\lambda_m,\lambda_1,\lambda_2] = \left(\frac{\alpha_1[\lambda_m]}{\nu_1[\lambda_m]+\delta+\sigma(\alpha_1[\lambda_1]\widehat{I}_{1,1}+\alpha_2\widehat{I}_{2,1}+\alpha_1[\lambda_2]\widehat{I}_{1,2}+\alpha_2\widehat{I}_{2,2})} + \frac{\nu_1[\lambda_m]}{\nu_1[\lambda_m]+\delta+\sigma(\alpha_1[\lambda_1]\widehat{I}_{1,1}+\alpha_2\widehat{I}_{2,1}+\alpha_1[\lambda_2]\widehat{I}_{1,2}+\alpha_2\widehat{I}_{2,2})}\frac{\alpha_2}{\nu_2+\delta}\right)\left(\widehat{S}+\sigma(\widehat{I}_{1,1}+\widehat{I}_{1,2})\right),$$

where $\widehat{I}_{1,1}, \widehat{I}_{1,2}, \widehat{I}_{2,1}, \widehat{I}_{2,2}$ depend upon λ_1 and λ_2.

If there is a stable coexistence equilibrium, we can follow the approach of Geritz et al. [5] to determine the long-term evolutionary dynamics after branching. These can be determined by examining the signs of $D_1(\lambda_1, \lambda_2) = \frac{\partial \mathcal{R}_0}{\partial \lambda_m}\Big|_{\lambda_m=\lambda_1}$ and $D_2(\lambda_1, \lambda_2) = \frac{\partial \mathcal{R}_0}{\partial \lambda_m}\Big|_{\lambda_m=\lambda_2}$ (this is because a rare mutant can only arise in either the type 1 or type 2 direction, but not both).

Here,

$$D_1(\lambda_1, \lambda_2) = \Bigg(-\frac{\nu_1'[\lambda_1]\,(\alpha_1[\lambda_1] + \nu_1[\lambda_1]k])}{(\nu_1[\lambda_1] + \delta + \sigma(\alpha_1[\lambda_1]\widehat{I}_{1,1} + \alpha_2\widehat{I}_{2,1} + \alpha_1[\lambda_2]\widehat{I}_{1,2} + \alpha_2\widehat{I}_{2,2}))^2} + \frac{\alpha_1'[\lambda_1] + \nu_1'[\lambda_1]k}{\nu_1[\lambda_1] + \delta + \sigma(\alpha_1[\lambda_1]\widehat{I}_{1,1} + \alpha_2\widehat{I}_{2,1} + \alpha_1[\lambda_2]\widehat{I}_{1,2} + \alpha_2\widehat{I}_{2,2})} \Bigg) \left(\widehat{S} + \sigma(\widehat{I}_{1,1} + \widehat{I}_{1,2})\right).$$

Note that by symmetry, $D_2(\lambda_1, \lambda_2) = D_1(\lambda_2, \lambda_1)$. Next, we note that $b_2 > c_2$ when there is a branching point, and we use an argument similar to that of Theorem 2 to prove the following result.

Theorem 1. *Suppose that there exists a branching point (so $b_2 > c_2$), and that there is a unique stable coexistence equilibrium. If there is a λ_i-isocline such that $D_i(\lambda_1, \lambda_2) = 0$, then it follows that it is a local minimum. Thus, if there exists an evolutionarily singular coalition $(\widehat{\lambda}_1, \widehat{\lambda}_2)$ such that $D_1(\widehat{\lambda}_1, \widehat{\lambda}_2) = 0$ and $D_2(\widehat{\lambda}_1, \widehat{\lambda}_2) = 0$, then it is evolutionarily unstable.*

Proof. The proof follows from an analogous argument to the proof of Theorem 2 and noting that

$$\frac{\partial}{\partial \lambda_m}\left(\frac{\partial \mathcal{R}_0[\lambda_m, \lambda_1, \lambda_2]}{\partial \lambda_m}\right)\Bigg|_{\lambda_m=\lambda_i} > 0.$$

□

Analysis for theorems and remarks in main text

First, as specified in the main text, we assume that $k = \frac{\alpha_2}{\nu_2+\delta} > 1$, *i.e.* the number of new infections that a fully symptomatic individual (in I_2) would have in a naive population is greater than one, which is biologically feasible. As in *Expanded Results*, *Electronic Supplementary Material*, $\gamma[\lambda] = \alpha_1[\lambda] + \nu_1[\lambda]k$.

Taking the partial derivative of $\mathcal{R}_0[\lambda, \lambda^*]$ in (6) with respect to the mutant phenotype λ^* gives

$$\frac{\partial \mathcal{R}_0}{\partial \lambda^*} = -\frac{\nu_1'[\lambda^*]}{(\nu_1[\lambda^*] + \sigma\gamma[\lambda]\widehat{I}_1[\lambda] + \delta)^2}(\alpha_1[\lambda^*] + \nu_1[\lambda^*]k)\left(\widehat{S}[\lambda] + \sigma\widehat{I}_1[\lambda]\right) + \frac{\alpha_1'[\lambda^*] + \nu_1'[\lambda^*]k}{\nu_1[\lambda^*] + \sigma\gamma[\lambda]\widehat{I}_1[\lambda] + \delta}\left(\widehat{S}[\lambda] + \sigma\widehat{I}_1[\lambda]\right).$$

Setting $D[\lambda] = \frac{\partial \mathcal{R}_0}{\partial \lambda^*}\Big|_{\lambda^*=\lambda} = 0$, multiplying by $(\nu_1[\lambda] + \sigma\gamma[\lambda]\widehat{I}_1[\lambda] + \delta)^2$ and rearranging gives

$$0 = \sigma\gamma[\lambda]\widehat{I}_1[\lambda](\alpha_1'[\lambda] + \nu_1'[\lambda]k) + \alpha_1'[\lambda]\delta + \nu_1'[\lambda]k\delta - \nu_1'[\lambda]\alpha_1[\lambda] + \nu_1[\lambda]\alpha_1'[\lambda].$$

Substituting the value of $\widehat{I}_1[\lambda]$ and $\gamma[\lambda]$, multiplying by $1 + \tau\nu_1[\lambda]$ where $\tau = \frac{1}{\nu_2+\delta} + \frac{1}{\mu+\delta}\frac{\nu_2}{\nu_2+\delta}$ gives that

$$\begin{aligned} 0 = {} & \sigma(\alpha_1'[\lambda] + \nu_1'[\lambda]k)(\alpha_1[\lambda] + \nu_1[\lambda]k - \nu_1[\lambda] - \delta) + \alpha_1'[\lambda]\delta + \tau\nu_1[\lambda]\alpha_1'[\lambda]\delta + \nu_1'[\lambda]k\delta \\ & + \tau\nu_1[\lambda]\nu_1'[\lambda]k\delta - \nu_1'[\lambda]\alpha_1[\lambda] - \tau\nu_1[\lambda]\nu_1'[\lambda]\alpha_1[\lambda] + \nu_1[\lambda]\alpha_1'[\lambda] + \tau\nu_1[\lambda]\nu_1[\lambda]\alpha_1'[\lambda] \end{aligned}$$

Substituting $\alpha_1[\lambda] = b_1 e^{-\lambda b_2} + \alpha_{1,\infty}$ and $\nu_1[\lambda] = c_1 e^{-\lambda c_2} + \nu_{1,\infty}$ into this equation and rearranging, we obtain

$$A_0 e^{-\lambda(2c_2+b_2)} + A_1 e^{-\lambda(2b_2)} + A_2 e^{-\lambda(2c_2)} + A_3 e^{-\lambda(b_2+c_2)} + A_4 e^{-\lambda b_2} + A_5 e^{-\lambda c_2} = 0. \quad (8)$$

Here, the coefficients are

$$\begin{aligned}
A_0 &= \tau c_1^2 b_1 (c_2 - b_2),\\
A_1 &= -b_1^2 b_2 \sigma,\\
A_2 &= c_1^2 c_2 (\tau(\alpha_{1,\infty} - k\delta) - \sigma k(k-1)),\\
A_3 &= b_1 c_1 (\tau c_2 \nu_{1,\infty} - 2\tau b_2 \nu_{1,\infty} - \delta\tau b_2 - b_2 + c_2 - (k-1) b_2 \sigma - k c_2 \sigma),\\
A_4 &= b_1 b_2 (-\tau \nu_{1,\infty}^2 - \delta\tau\nu_{1,\infty} - \nu_{1,\infty} - \alpha_{1,\infty}\sigma - (k-1)\nu_{1,\infty}\sigma - \delta + \delta\sigma),\\
A_5 &= c_1 c_2 (\tau\alpha_{1,\infty}\nu_{1,\infty} - \tau k\delta\nu_{1,\infty} + \alpha_{1,\infty} - k\delta - \alpha_{1,\infty} k\sigma - k(k-1)\nu_{1,\infty}\sigma + \delta k\sigma).
\end{aligned}$$

Multiplying (8) by $e^{\lambda(2c_2+2b_2)}$ gives

$$A_0 e^{\lambda b_2} + A_1 e^{\lambda(2c_2)} + A_2 e^{\lambda(2b_2)} + A_3 e^{\lambda(b_2+c_2)} + A_4 e^{\lambda(2c_2+b_2)} + A_5 e^{\lambda(2b_2+c_2)} = 0, \quad (9)$$

which is equivalent to $D[\lambda] = 0$. It is obvious that $A_1 < 0$. Furthermore, the signs of A_2 and A_5 are determined by conditions on $\alpha_{1,\infty}$.

Theorem 2. *If $b_2 > c_2$, then any interior evolutionarily singular strategy is a fitness minimum and is thus not ESS. If $c_2 > b_2$, then any interior evolutionarily singular strategy is an ESS. Furthermore, by definition, if there exists a boundary attractor then it is an ESS.*

Proof. Evolutionarily singular strategies are so that $D[\lambda] = \left.\frac{\partial \mathcal{R}_0}{\partial \lambda^*}\right|_{\lambda^*=\lambda} = 0$. To find if they are evolutionarily stable, we require the sign of $\left.\frac{\partial}{\partial \lambda^*}\left(\frac{\partial \mathcal{R}_0[\lambda,\lambda^*]}{\partial \lambda^*}\right)\right|_{\lambda^*=\lambda}$ and so we compute

$$\begin{aligned}
\frac{\partial}{\partial \lambda^*}\left(\frac{\partial \mathcal{R}_0[\lambda,\lambda^*]}{\partial \lambda^*}\right) = \Bigg(& \frac{2\nu_1'[\lambda^*]\nu_1'[\lambda^*]\left(\alpha_1[\lambda^*] + \nu_1[\lambda^*]k\right)}{(\nu_1[\lambda^*] + \sigma\gamma[\lambda]\widehat{I}_1[\lambda] + \delta)^3} - \frac{2\nu_1'[\lambda^*](\alpha_1'[\lambda^*] + \nu_1'[\lambda^*]k)}{(\nu_1[\lambda^*] + \sigma\gamma[\lambda]\widehat{I}_1[\lambda] + \delta)^2}\\
& - \frac{\nu''[\lambda^*](\alpha_1[\lambda^*] + \nu_1[\lambda^*]k)}{(\nu_1[\lambda^*] + \sigma\gamma[\lambda]\widehat{I}_1[\lambda] + \delta)^2} + \frac{\alpha_1''[\lambda^*] + \nu_1''[\lambda^*]k}{\nu_1[\lambda^*] + \sigma\gamma[\lambda]\widehat{I}_1[\lambda] + \delta}\Bigg)\left(\widehat{S}[\lambda] + \sigma\widehat{I}_1[\lambda]\right).
\end{aligned}$$

Evaluating this at $\lambda^* = \lambda$ and noting that $-\frac{\nu_1'[\lambda](\alpha_1[\lambda]+\nu_1[\lambda]k])}{(\nu_1[\lambda]+\sigma\gamma[\lambda]\widehat{I}_1[\lambda]+\delta)^2} + \frac{\alpha_1'[\lambda]+\nu_1'[\lambda]k}{\nu_1[\lambda]+\sigma\gamma[\lambda]\widehat{I}_1[\lambda]+\delta} = 0$ since λ is an evolutionarily singular strategy, it follows

$$\left.\frac{\partial}{\partial \lambda^*}\left(\frac{\partial \mathcal{R}_0[\lambda,\lambda^*]}{\partial \lambda^*}\right)\right|_{\lambda^*=\lambda} = \left(-\frac{\nu_1''[\lambda](\alpha_1[\lambda] + \nu_1[\lambda]k)}{(\nu_1[\lambda] + \sigma\gamma[\lambda]\widehat{I}_1[\lambda] + \delta)^2} + \frac{\alpha_1''[\lambda] + \nu_1''[\lambda]k}{\nu_1[\lambda] + \sigma\gamma[\lambda]\widehat{I}_1[\lambda] + \delta}\right)\left(\widehat{S}[\lambda] + \sigma\widehat{I}_1[\lambda]\right). \quad (10)$$

It also follows that $\nu_1''[\lambda] = -c_2\nu_1'[\lambda]$, and that $\alpha_1''[\lambda] = -b_2\alpha_1'[\lambda]$. Substituting these in the above give

$$\left.\frac{\partial}{\partial \lambda^*}\left(\frac{\partial \mathcal{R}_0[\lambda,\lambda^*]}{\partial \lambda^*}\right)\right|_{\lambda^*=\lambda} = \left(\frac{c_2\nu_1'[\lambda](\alpha_1[\lambda] + \nu_1[\lambda]k)}{(\nu_1[\lambda] + \sigma\gamma[\lambda]\widehat{I}_1[\lambda] + \delta)^2} - \frac{b_2\alpha_1'[\lambda] + c_2\nu_1'[\lambda]k}{\nu_1[\lambda] + \sigma\gamma[\lambda]\widehat{I}_1[\lambda] + \delta}\right)\left(\widehat{S}[\lambda] + \sigma\widehat{I}_1[\lambda]\right). \quad (11)$$

Since $\alpha_1'[\lambda] < 0$ by definition, it follows that at an evolutionarily singular strategy,

$$\frac{\partial}{\partial\lambda^*}\left(\frac{\partial\mathcal{R}_0[\lambda,\lambda^*]}{\partial\lambda^*}\right)\Bigg|_{\lambda^*=\lambda} > c_2\left(\frac{\nu_1'[\lambda](\alpha_1[\lambda]+\nu_1[\lambda]k)}{(\nu_1[\lambda]+\sigma\gamma[\lambda]\widehat{I}_1[\lambda]+\delta)^2}-\frac{\alpha_1'[\lambda]+\nu_1'[\lambda]k}{\nu_1[\lambda]+\sigma\gamma[\lambda]\widehat{I}_1[\lambda]+\delta}\right)\left(\widehat{S}[\lambda]+\sigma\widehat{I}_1[\lambda]\right)=0 \quad (12)$$

if and only if $b_2 > c_2$, which completes the first part of the proof.

The second part of the proof follows immediately from the definition of an ESS. Indeed, for a strategy $\widehat{\lambda}$ to be ESS, it is required that no mutant can invade a resident with such a strategy. If there is a local attractor at the $\widehat{\lambda} = 0$ boundary, then $D[0] < 0$, and so no mutants $\lambda^* > 0$ can invade. Since the mutant phenotype space is $\lambda^* \in [0, \infty)$, then it follows by definition that $\widehat{\lambda} = 0$ is a local ESS. Second, suppose there is a local attractor at the $\widehat{\lambda} = \infty$ boundary. Then, there is some large enough $\lambda_{\min}$ such that $D[\lambda] > 0$ for all $\lambda > \lambda_{\min}$. Thus, $\widehat{\lambda} = \infty$ is a local ESS.

If the formulations of $\alpha_1[\lambda]$ and $\nu_1[\lambda]$ are more general and instead take the form of Eqs. (4–5), i.e. $\alpha_1[\lambda] = b_1(F[\lambda])^{-b_2} + \alpha_{1,\infty}$ and $\nu_1[\lambda] = c_1(F[\lambda])^{-c_2} + \nu_{1,\infty}$, then

$$\alpha_1''[\lambda] = \alpha_1'[\lambda]\left(-(b_2+1)\frac{F'[\lambda]}{F[\lambda]}+\frac{F''[\lambda]}{F'[\lambda]}\right),$$

$$\nu_1''[\lambda] = \nu_1'[\lambda]\left(-(c_2+1)\frac{F'[\lambda]}{F[\lambda]}+\frac{F''[\lambda]}{F'[\lambda]}\right).$$

Using these relations in Eq. (10) and noting that $F[\lambda] > 0, F'[\lambda] > 0$ (by definition of $F[\lambda]$) gives the same result.

□

Remark 1. *Let $\alpha_{1,0} = \alpha_1[0] = b_1 + \alpha_{1,\infty}$ and $\nu_{1,0} = \nu_1[0] = c_1 + \nu_{1,\infty}$. It can be shown that $\sum_{i=1}^5 A_i > 0$ iff*

$$-\sigma\left(\frac{b_1b_2}{c_1c_2}+k\right)(\alpha_{1,0}+(k-1)\nu_{1,0}-\delta)+(1+\tau\nu_{1,0})\left((\alpha_{1,0}-k\delta)-\frac{b_1b_2}{c_1c_2}(\nu_{1,0}+\delta)\right)>0.$$

Remark 2. *The single strain basic reproduction number is greater than 1. At infinity, this is $\frac{\alpha_{1,\infty}+k\nu_{1,\infty}}{\nu_{1,\infty}+\delta} > 1$. Thus, the inequality $\delta < \alpha_{1,\infty} + (k-1)\nu_{1,\infty}$ must be satisfied, which gives that $A_4 < 0$. Furthermore, since we assume that the single strain basic reproduction number is greater than 1 for all $\alpha_{1,\infty}$, it follows that $\delta < (k-1)\nu_{1,\infty}$.*

Theorem 3. *Assume that $b_2 > c_2$.*

(S1) Suppose that $A_2 < 0$ and $A_5 < 0$. Then, there is a unique ESS at $\widehat{\lambda} = 0$.

(S2) Suppose that $A_2 < 0$ and $A_5 > 0$. Then, if $\sum_{i=1}^5 A_i < 0$, there is a unique evolutionarily singular strategy that is not convergence stable. Since this strategy is not-ESS by Theorem 1, then it leads to two bistable local ESSs at $\widehat{\lambda} = 0$ and $\widehat{\lambda} = \infty$. If $\sum_{i=1}^5 A_i > 0$ then $D[\lambda]$ is an increasing function of λ and the unique ESS is at $\widehat{\lambda} = \infty$.

(S3) Suppose that $A_2 > 0$ and $A_5 > 0$. Then, if $\sum_{i=1}^5 A_i < 0$, there is at least one evolutionarily singular strategy that is not convergence stable, and leads to alternative stable states since the singular strategy is not an ESS (by Theorem 1). Otherwise, if $\sum_{i=1}^5 A_i > 0$, then there is a local ESS at $\widehat{\lambda} = \infty$ and there are possibly other interior singular strategies. If $b_2 > 2c_2$, then the interior evolutionarily singular strategy is unique if $\sum_{i=1}^5 A_i < 0$ and the ESS at infinite latency is unique if $\sum_{i=1}^5 A_i > 0$.

(S4) Suppose that $A_2 > 0$ and $A_5 < 0$. Then, if $\sum_{i=1}^{5} A_i > 0$, there is a unique interior convergence stable singular strategy $\widehat{\lambda} > 0$. Since this strategy is not an ESS by Theorem 1, then it is a branching point and leads to mutual invasibility of strategies with different latencies. Otherwise, if $\sum_{i=1}^{5} A_i < 0$, there is a local ESS at $\widehat{\lambda} = 0$, and there are possibly other interior singular strategies.

Proof. First, we prove (S1). Since $b_2 > c_2$, it follows that $A_0 < 0$, and $A_3 < 0$ also since the sum of the first two terms of A_3 are negative if $c_2 < 2b_2$ and the fourth and fifth term are also negative if $c_2 < b_2$. Since $A_2 < 0$ and $A_5 < 0$, this gives that $A_i < 0$ for all i. Thus, $D[\lambda] < 0$ for all λ, and the unique ESS is at $\widehat{\lambda} = 0$.

Next, we prove (S2), and assume that $A_2 < 0$ and $A_5 > 0$. Then, rearranging this equation and multiplying by $e^{-\lambda(2b_2+c_2)}$ gives

$$-A_0 e^{-\lambda(b_2+c_2)} - A_1 e^{-\lambda(2b_2-c_2)} - A_2 e^{-\lambda c_2} - A_3 e^{-\lambda b_2} - A_4 e^{-\lambda(b_2-c_2)} = A_5.$$

Clearly, the left-hand side is a decreasing function, and so if $\sum_{i=1}^{5} A_i < 0$, there is a unique intersection and thus a unique root which is an evolutionarily singular strategy. Since $\sum_{i=1}^{5} A_i < 0$, then $D[\lambda] < 0$ for λ less than the evolutionarily singular strategy. Furthermore, since $A_5 > 0$, this implies that $D[\lambda]$ is positive as λ approaches ∞, *i.e.* $D[\lambda] > 0$ for λ greater than the evolutionarily singular strategy. Thus, according to Brannstrom et al. [6], this interior singular strategy is not convergence stable. This leads to bistability at $\widehat{\lambda} = 0$ and $\widehat{\lambda} = \infty$. Otherwise, if $\sum_{i=1}^{5} A_i > 0$, there are no interior roots. If there are no interior evolutionarily singular strategies, then the unique ESS is at $\widehat{\lambda} = \infty$ since $D[\lambda] > 0$.

Now, we prove statement (S3), so we suppose that $A_2 > 0$ and $A_5 > 0$, and note that $A_3 < 0$ since $b_2 > c_2$ We also first assume that $b_2 > 2c_2$. Then, rearranging (9) and multiplying by $e^{-\lambda(2b_2)}$ gives

$$-A_0 e^{-\lambda b_2} - A_1 e^{-\lambda(2(b_2-c_2))} - A_3 e^{-\lambda(b_2-c_2)} - A_4 e^{-\lambda(b_2-2c_2)} = A_5 e^{\lambda c_2} + A_2.$$

It is clear that the left-hand side is a decreasing function of λ, whereas the right-hand side is an increasing function of λ, from $A_5 + A_2$ to ∞. Thus, if $\sum_{i=1}^{5} A_i < 0$, there is a unique root and so a unique evolutionarily singular strategy that is not convergent stable (as in the previous case). This singular strategy thus gives rise to bistability at $\widehat{\lambda} = 0$ and at $\widehat{\lambda} = \infty$ since $A_5 > 0$. Otherwise, if $\sum_{i=1}^{5} A_i > 0$, there are no roots and no interior evolutionarily singular strategies, and $\widehat{\lambda} = \infty$ is the unique ESS.

Suppose now that $b_2 < 2c_2$. Since $A_5 > 0$, then $D[\lambda] > 0$ for large λ, so there is an attracting state at $\widehat{\lambda} = \infty$. If $\sum_{i=1}^{5} A_i < 0$, then $D[0] < 0$. Thus, there is least one interior evolutionarily singular strategy that is not convergence stable (as previously). If $\sum_{i=1}^{5} A_i > 0$, then $D[0] > 0$, and so in addition to the local attracting state at $\widehat{\lambda} = \infty$ (since $D[\lambda]$ is positive as λ approaches infinity), there might be other interior evolutionarily singular strategies if $D[\lambda]$ is not monotonic.

Lastly, we prove (S4), so $A_2 > 0$ and $A_5 < 0$. If $\sum_{i=1}^{5} A_i > 0$, then there is at least one interior singular strategy that is convergence stable since $D[0] > 0$ and $D[\lambda] < 0$ for large enough λ. To prove uniqueness, a more sophisticated argument to the ones used previously is required, as the function $f[\lambda]$ defined by the LHS of (9) cannot be separated into separate increasing and decreasing functions. However, noting that $A_0, A_1, A_3, A_4, A_5 < 0$ and that A_5 is the coefficient of the term with the largest exponent implies that there are exactly two sign changes in the coefficient of the LHS of (9). According to Jameson [7] (who attributes this to Laguerre), this implies that $f[\lambda]$ has at most two positive zeros, since $f[\lambda]$ can be viewed as a 'generalized polynomial', *i.e.* consider the transformation $x = e^{\lambda}$. Since $A_5 < 0$ and we are assuming that $\sum_{i=1}^{5} A_i > 0$, then $f[0]$ and $f[\infty]$ have different signs, which implies that there is an odd positive number of roots to $f[\lambda] = 0$. Therefore, it follows that there is a unique root to $f[\lambda]$ and so the singular strategy is unique in this case. For the more general

formulations with $F[\lambda]$, we note that a similar argument follows also, by considering $x = F[\lambda]$ and noting that $x > 0$. Since this singular strategy is not ESS, then this singular strategy gives mutual invasibility of nearby types through branching.

Otherwise, if $\sum_{i=1}^{5} A_i < 0$, then there is a local attracting state at $\widehat{\lambda} = 0$, and there are possibly other interior singular strategies. □

Remark 3. *In Theorem 3, i.e. when $b_2 > c_2$, the conditions on A_2 and A_5 are conditions on $\frac{\alpha_{1,\infty}}{\delta}$. Let $\mathcal{B}_1 = k + \frac{\sigma k(k-1)}{\tau\delta}$ and $\mathcal{B}_2 = k + \frac{\sigma k(k-1)(\nu_{1,\infty}+\delta)/\delta}{\tau\nu_{1,\infty}+1-k\sigma}$, as the notation used in Figure 2 and Expanded Results, Electronic Supplementary Material. First, note that if $\sigma > \frac{1+\tau\nu_{1,\infty}}{k}$, then $A_5 > 0$ if and only if $\frac{\alpha_{1,\infty}}{\delta} < \mathcal{B}_2$, which implies that $A_2 < 0$ since $\mathcal{B}_2 < \mathcal{B}_1$ and $\frac{\alpha_{1,\infty}}{\delta} < \mathcal{B}_1$. Consider the following inequalities on the superinfection parameter σ:*

(S1) $\sigma > \frac{1+\tau\nu_{1,\infty}}{k}$:

- *(a) $\frac{\alpha_{1,\infty}}{\delta} < \mathcal{B}_2 < \mathcal{B}_1 \implies A_2 < 0, A_5 > 0$,*
- *(b) $\mathcal{B}_2 < \frac{\alpha_{1,\infty}}{\delta} < \mathcal{B}_1 \implies A_2 < 0, A_5 < 0$,*
- *(c) $\mathcal{B}_2 < \mathcal{B}_1 < \frac{\alpha_{1,\infty}}{\delta} \implies A_2 > 0, A_5 < 0$,*

(S2) $\sigma < \frac{1-\tau\delta}{k}$ (then $\mathcal{B}_1 > \mathcal{B}_2 > 0$):

- *(a) $\frac{\alpha_{1,\infty}}{\delta} < \mathcal{B}_2 < \mathcal{B}_1 \implies A_2 < 0, A_5 < 0$,*
- *(b) $\mathcal{B}_2 < \frac{\alpha_{1,\infty}}{\delta} < \mathcal{B}_1 \implies A_2 < 0, A_5 > 0$,*
- *(c) $\mathcal{B}_2 < \mathcal{B}_1 < \frac{\alpha_{1,\infty}}{\delta} \implies A_2 > 0, A_5 > 0$,*

(S3) $\frac{1+\tau\nu_{1,\infty}}{k} > \sigma > \frac{1-\tau\delta}{k}$ (then $\mathcal{B}_2 > \mathcal{B}_1 > 0$):

- *(a) $\frac{\alpha_{1,\infty}}{\delta} < \mathcal{B}_1 < \mathcal{B}_2 \implies A_2 < 0, A_5 < 0$,*
- *(b) $\mathcal{B}_1 < \frac{\alpha_{1,\infty}}{\delta} < \mathcal{B}_2 \implies A_2 > 0, A_5 < 0$,*
- *(c) $\mathcal{B}_1 < \mathcal{B}_2 < \frac{\alpha_{1,\infty}}{\delta} \implies A_2 > 0, A_5 > 0$,*

Remark 4. *We can rewrite $\mathcal{B}_2$ as $\mathcal{B}_2 = k\frac{\delta(\tau\nu_{1,\infty}+1)+\sigma((k-1)\nu_{1,\infty}-\delta)}{\delta(\tau\nu_{1,\infty}+1-k\sigma)}$. If $\sigma > \frac{1+\tau\nu_{1,\infty}}{k}$, then the denominator is negative and so $\mathcal{B}_2 > 0$ if and only if the numerator is also negative, i.e. $\delta(\tau\nu_{1,\infty}+1) + \sigma((k-1)\nu_{1,\infty} - \delta) < 0$. But $\delta < (k-1)\nu_{1,\infty}$ by Remark 2 and so this is impossible, thus $\mathcal{B}_2 < 0$ whenever $\sigma > \frac{1+\tau\nu_{1,\infty}}{k}$.*

Theorem 4. *Suppose that $c_2 > b_2$.*

(S1) If $\sum_{i=1}^{5} A_i > 0$, then there is at least one interior convergence stable singular strategy that is ESS, and possibly other interior singular strategies. If $\sum_{i=1}^{5} A_i < 0$, then there is a local attracting state at $\widehat{\lambda} = 0$, and possibly other interior singular strategies.

(S2) The convergence stable interior singular strategy or the attracting state at $\widehat{\lambda} = 0$ is unique in the following cases:

- *(a) $A_2 > 0$, $A_5 < 0$,*
- *(b) $A_2, A_3, A_5 < 0$,*
- *(c) $A_2, A_5 > 0$, $c_2 > b_2(\max\{2, \frac{2\tau\nu_{1,\infty}+\delta\tau+1+(k-1)\sigma}{\tau\nu_{1,\infty}+1-k\sigma}\})$.*

Proof. First, we prove the first statement, which is more general. The condition that $c_2 > b_2$ implies that the largest exponent is $2c_2 + b_2$ in (9), with corresponding coefficient A_4. Since $A_4 < 0$, it follows that if $\sum_{i=1}^{5} A_i > 0$, then there is at least one root to (9). Noting that $D[0] > 0$ and $D[\lambda]$ is negative as λ approaches

infinity, it follows that this root is convergence stable (as in Theorem 2). If $\sum_{i=1}^{5} A_i < 0$, then this implies that $D[0]$ is negative, thus giving that $\widehat{\lambda} = 0$ is a local attracting state. Since $c_2 > b_2$, then Theorem 1 gives that this interior singular strategy is an ESS.

Now, we prove the three cases in the second statement. First, note that $c_2 > b_2$ implies that $b_2 < 2b_2 < b_2 + c_2 < 2b_2 + c_2, 2c_2 < 2c_2 + b_2$, where the ordering of $2b_2 + c_2$ and $2c_2$ is dictated by the relative magnitudes of $2b_2$ and c_2. For (a), first assume that $A_3 < 0$. Then, rearranging (9) and multiplying by $e^{-\lambda(2b_2)}$ gives

$$A_0 e^{-\lambda b_2} + A_2 = -A_1 e^{\lambda(2(c_2-b_2))} - A_3 e^{\lambda(c_2-b_2)} - A_4 e^{\lambda(2c_2-b_2)} - A_5 e^{\lambda c_2}.$$

It is clear that the right-hand side is an increasing function of λ to infinity, whereas the left-hand side is a strictly decreasing function of λ. Therefore, there is exactly one positive root if $\sum_{i=1}^{5} A_i > 0$, otherwise there are no positive roots to this equation. Likewise, if $A_3 > 0$, rearranging (9) and multiplying by $e^{-\lambda(b_2+c_2)}$ gives

$$A_0 e^{-\lambda c_2} + A_2 e^{-\lambda(c_2-b_2)} + A_3 = -A_1 e^{\lambda(c_2-b_2)} - A_4 e^{\lambda c_2} - A_5 e^{\lambda b_2}.$$

Again, it is clear that the left-hand side is a decreasing function whereas the right-hand side is an increasing function to infinity, and so there is exactly one positive root if and only if $\sum_{i=1}^{5} A_i > 0$. Thus, the sign of A_3 is irrelevant under the conditions that $A_2 > 0$ and $A_5 < 0$: there is exactly one positive root if $\sum_{i=1}^{5} A_i > 0$ and none if $\sum_{i=1}^{5} A_i < 0$. Furthermore, if there is a root, it is the unique convergence stable strategy, and if there are none, then the unique attracting state is at $\widehat{\lambda} = 0$. For (b), rewriting (9) and multiplying by $e^{-\lambda b_2}$ gives

$$A_0 = -A_1 e^{\lambda(2c_2-b_2)} - A_2 e^{\lambda b_2} - A_3 e^{\lambda c_2} - A_4 e^{\lambda(2c_2)} - A_5 e^{\lambda(b_2+c_2)}.$$

As previously, the right-hand side is an increasing function, and so there is exactly one positive root if $\sum_{i=1}^{5} A_i > 0$, which is the unique convergence stable singular strategy. Otherwise, if $\sum_{i=1}^{5} A_i < 0$, then there are no positive roots and the attracting state is at $\widehat{\lambda} = 0$. For (c), rewriting (9) and multiplying by $e^{-\lambda(2b_2+c_2)}$ gives

$$A_0 e^{-\lambda(c_2+b_2)} + A_2 e^{-\lambda c_2} + A_3 e^{-\lambda b_2} + A_5 = -A_1 e^{\lambda(c_2-2b_2)} - A_4 e^{\lambda(c_2-b_2)}.$$

Here, we note that the third condition gives $c_2 > 2b_2$, in addition to ensuring $A_3 > 0$ (solving for c_2 in terms of b_2 gives the second value in the maximum equation). Thus, the left-hand side is a decreasing function, whereas the right-hand side is an increasing function, and there is exactly one positive root if $\sum_{i=1}^{5} A_i > 0$, and this is the globally attracting state. Otherwise, if $\sum_{i=1}^{5} A_i < 0$, then there is no root, and $D[\lambda] < 0$ for all λ the global attractor is therefore at $\widehat{\lambda} = 0$. □

Remark 5. *In the proofs of the theorems, we have assumed the exponential formulation of the trade-offs for simplicity. Suppose instead that the more general form presented in the main text is considered. Note that we assume $F'[\lambda] > 0$, thus, $F'[\lambda]$ is a factor in every term of $D[\lambda]$. Therefore, setting $D[\lambda] = 0$ gives an equivalent relation as in Eq. (9), with the substitution of e^{λ} by $F[\lambda]$ sufficing to prove equivalent results.*

The superinfection parameter as a function of latency

We have assumed that the superinfection parameter σ is constant, but it is possible that a strain with higher latency would have a smaller value of σ. As a simplest case, we model σ as $\sigma[\lambda] = d_3[d_1 e^{-d_2\lambda} + (1 - d_1)]$. Thus,

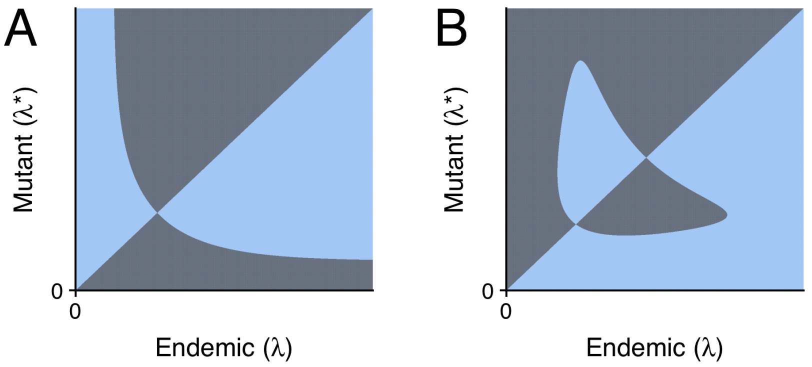

Figure S1: The effect of the superinfection parameter decreasing as a function of latency λ. Here, we assume that $\sigma[\lambda] = d_3[d_1 e^{-d_2\lambda} + (1-d_1)]$ for $d_2 \geq 0$. (*A*) is as in the middle panel of Fig. 4C, i.e. $d_2 = 0$, whereas (*B*) $d_2 = 1.05$. In both (*A*) and (*B*), $d_1 = d_3 = 1$.

with $\sigma = \sigma[\lambda]$ and $\sigma^* = \sigma[\lambda^*]$, Equations (2) become

$$\frac{dI_1^*}{dt} = \alpha_1^* I_1^* S + \alpha_2 I_2^* S + \sigma^* \alpha_1^* I_1 I_1^* + \sigma^* \alpha_2 I_1 I_2^* - (\nu_1^* + \delta) I_1^* - \sigma(\alpha_1 I_1 I_1^* + \alpha_2 I_2 I_1^*), \tag{13}$$

$$\frac{dI_2^*}{dt} = \nu_1^* I_1^* - (\nu_2 + \delta) I_2^*,$$

and so the invading reproduction number is now

$$\mathcal{R}_0[\lambda, \lambda^*] = \left(\frac{\alpha_1[\lambda^*]}{\nu_1[\lambda^*] + \delta + \sigma[\lambda](\alpha_1[\lambda]\widehat{I}_1[\lambda] + \alpha_2 \widehat{I}_2[\lambda])} + \frac{\nu_1[\lambda^*]}{\nu_1[\lambda^*] + \delta + \sigma[\lambda](\alpha_1[\lambda]\widehat{I}_1[\lambda] + \alpha_2 \widehat{I}_2[\lambda])} \frac{\alpha_2}{\nu_2 + \delta} \right) \left(\widehat{S}[\lambda] + \sigma[\lambda^*]\widehat{I}_1[\lambda] \right), \tag{14}$$

With $d_1 = d_3 = 1$, we present in Fig. S1 the effect of increasing d_2. Fig. S1A has $d_2 = 0$ and is identical to Fig. 4C (*middle panel*). On the other hand, $d_2 = 1.05$ in Fig S1B and there is an interior evolutionarily singular strategy that is not an ESS. However, with a constant superinfection parameter, we have proved in Theorem 2 that any interior evolutionarily singular strategy is ESS. Thus, the dependence of σ on λ introduces additional possible evolutionary behavior.

References

[1] H Guo, MY Li, Global dynamics of a staged progression model for infectious diseases. *Mathematical Biosciences and Engineering* **3**, 513–525 (2006).

[2] G Röst, T Tekeli, Stability and oscillations of multistage sis models depend on the number of stages. *Applied Mathematics and Computation* **380**, 125259 (2020).

[3] O Diekmann, JAP Heesterbeek, JAJ Metz, On the definition and the computation of the basic reproduction ratio R_0 in models for infectious diseases in heterogeneous populations. *Journal of Mathematical Biology* **28**, 365–382 (1990).

[4] P van den Driessche, J Watmough, Reproduction numbers and sub-threshold endemic equilibria for compartmental models of disease transmission. *Mathematical Biosciences* **180**, 29 – 48 (2002).

[5] SAH Geritz, E Kisdi, G Meszéna, JAJ Metz, Evolutionarily singular strategies and the adaptive growth and branching of the evolutionary tree. *Evolutionary Ecology* **12**, 35–57 (1998).

[6] A Brannstrom, J Johansson, N Von Festenberg, The hitchhiker's guide to adaptive dynamics. *Games* **4**, 304–328 (2013).

[7] G Jameson, The number of zeros of a sum of fractional powers. *Proceedings of the Royal Society A: Mathematical, Physical and Engineering Sciences* **462**, 1821–1830 (2006).
